# Idiopathic Pulmonary Fibrosis Mortality by Industry and Occupation — United States, 2020–2022

**DOI:** 10.15585/mmwr.mm7407a1

**Published:** 2025-03-06

**Authors:** Jacek M. Mazurek, Girija Syamlal, David N. Weissman

**Affiliations:** 1 Respiratory Health Division, National Institute for Occupational Safety and Health, CDC.

**Summary**

**What is already known about this topic?**

Idiopathic pulmonary fibrosis (IPF), a progressive lung disease characterized by scarring and worsening lung function, has a poor prognosis. An estimated 21% of IPF deaths might be attributable to occupational exposures.

**What is added by this report?**

This exploratory analysis of 2020–2022 multiple cause-of-death data identified 67,843 IPF deaths among U.S. workers. By industry, IPF mortality was most significantly elevated among males employed in utilities and among females employed in public administration. By occupation, IPF mortality was most significantly elevated among male community and social services workers and among female farming, fishing, and forestry workers.

**What are the implications for public health practice?**

Estimates of elevated IPF mortality among workers in some industries and occupations warrant confirmation and continued surveillance to identify occupational exposures that could be targeted to prevent or reduce IPF mortality.

## Abstract

Idiopathic pulmonary fibrosis (IPF), a progressive lung disease characterized by scarring and worsening lung function, has a poor prognosis. A recent systematic review estimated that 21% of IPF deaths might be attributable to occupational exposures. To describe IPF mortality among U.S. residents aged ≥15 years who were ever employed, by industry and occupation, CDC conducted an exploratory analysis of 2020–2022 multiple cause-of-death data. During 2020–2022, a total of 67,843 (39,712 [59%] male and 28,131 [41%] female) decedents had IPF, suggesting that during this 3-year period, 8,340 IPF deaths in males and 5,908 deaths in females might have been associated with occupational exposures. By industry group, the highest proportionate mortality ratios among males were among those employed in utilities (1.15) and among females, were among those employed in public administration (1.12). By occupation group, the highest IPF mortality rates among males were among community and social services workers (1.23) and among females among farming, fishing, and forestry workers (1.24). Estimates of elevated IPF mortality among workers in specific industries and occupations warrant confirmation, control of known exposure-related risk factors, and continued surveillance to better understand the full range of occupational exposures that might increase risk for developing IPF. 

## Introduction

Idiopathic pulmonary fibrosis (IPF) is characterized by progressive scarring of lung tissue and declining lung function, with a median survival of 3–5 years after diagnosis (*1*). Typically, the disease presents with unexplained, progressive shortness of breath, often accompanied by a nonproductive cough and a radiographic pattern of usual interstitial pneumonia on high-resolution computed tomography (*1*,*2*). Treatment options are noncurative and include pharmacotherapies (i.e., pirfenidone and nintedanib to slow progression) and lung transplantation (*1*,*2*).

In 2017, the overall IPF age-adjusted death rate (IPF deaths per 100,000 persons) was 5.4 (7.2 for men and 4.0 for women) (*3*). Although the exact IPF etiology remains unknown, studies have indicated associations with cigarette smoking, genetic mutations, viral infections (e.g., Epstein-Barr virus and hepatitis C), and occupational exposures to pesticides, and wood (pine) and metal (brass, lead, and steel) dust (*1*,*2*,*4*,*5*). The proportion of IPF cases associated with occupational exposures (the population attributable fraction) has been previously estimated to be 21% (95% CI = 15%–28%)* (*6*). To describe IPF mortality among U.S. residents aged ≥15 years by industry and occupation and to examine associations among IPF deaths and industry and occupation, CDC analyzed 2020–2022 multiple cause-of-death data.

## Methods

### Data Source

Deaths were identified from the National Vital Statistics System’s (NVSS) public-use multiple cause-of-death files,^†^ which include 10,038,112 records for U.S. residents aged ≥15 years who died during 2020–2022, the most recent years the jurisdictions^§^ provided information on decedents’ usual^¶^ industry and occupation. The NVSS mortality files include coded industry (23 major groups) and occupation (26 major groups) information for 9,738,271 decedents.**

### Case Definition

IPF decedents were defined as persons whose death record listed the *International Classification of Diseases, Tenth Revision* (ICD-10) code J84.1 (other interstitial pulmonary diseases with fibrosis) as the underlying^††^ or a contributing cause of death. Following previous reports (*3*), death records listing ICD-10 codes for conditions that might be associated with nonidiopathic pulmonary fibrosis^§§^ were excluded from the analysis (3,033 [4.3%] of 70,876 death certificates listing ICD-10 code J84.1).

### Data Analyses

Death rates (deaths per 100,000 persons aged ≥15 years) were based on postcensal population estimates as of July 1 of the corresponding year and were age-adjusted using the 2000 U.S. Census Bureau standard population.^¶¶^ Because some occupations are dominated by either male (e.g., mechanics, carpenters, and electricians) or female (e.g., preschool and kindergarten teachers, childcare workers, and administrative assistants) workers,*** and because the IPF-related mortality is higher in males than in females (*3*), proportionate mortality ratios (PMRs),^†††^ adjusted by 10-year age groups, race and ethnicity, and 95% CIs assuming Poisson distribution of the data were calculated by industry and occupation for males and females. One occupation might be listed under multiple industries. Analyses were conducted using SAS software (version 9.4; SAS Institute). This activity was reviewed by CDC, deemed not research, and was conducted consistent with applicable federal law and CDC policy.^§§§^

## Results

### IPF Deaths and Death Rates

During 2020–2022, a total of 67,843 deaths with IPF listed as the underlying (38,869; 57.3%) or a contributing (28,974; 42.7%) cause of death among U.S. residents aged ≥15 years were reported (Table 1), accounting for 0.7% of 9,738,271 deaths from all causes. Among IPF deaths, 45,646 (67.3%) occurred among persons aged ≥75 years, 39,712 (58.5%) occurred among males, 61,356 (90.4%) among White persons, and 60,793 (89.6%) among non-Hispanic persons. Overall, the annualized age-adjusted IPF death rate was 7.1 per 100,000 persons. The highest IPF death rates were among adults aged ≥75 years (67.6 per 100,00 persons), males (7.7), non-Hispanic (7.7), and White persons (8.2). Based on an estimate that 21% of IPF deaths might be related to occupational exposures, during this 3-year period, 8,340 (95% CI = 5,957–11,119) IPF deaths in males and 5,908 (95% CI = 4,220–7,877) deaths in females might have resulted from occupational exposures.

**TABLE 1:** Characteristics of idiopathic pulmonary fibrosis decedents* and age-adjusted idiopathic pulmonary fibrosis death rates^†^ among ever-employed^§^ persons aged ≥15 years, by sex (N = 67,843) — selected U.S. jurisdictions,^¶^ 2020–2022

Characteristic	Males		Females		Total	
	No. of deaths (% of IPF deaths)	Death rate (95% CI)	No. of deaths (% of IPF deaths)	Death rate (95% CI)	No. of deaths (% of IPF deaths)	Death rate (95% CI)
**Total (% of all IPF deaths)**	**39,712 (58.5)**	**7.7 (7.6–7.9)**	**28,131 (41.5)**	**6.2 (6.0–6.3)**	**67,843 (100.0)**	**7.1 (7.0–7.2)**
Age group, yrs**						
15–34	74 (0.2)	0.06 (0.05–0.07)	67 (0.2)	0.05 (0.04–0.07)	**141 (0.2)**	**0.05 (0.04–0.06)**
35–44	165 (0.4)	0.26 (0.2–0.3)	148 (0.5)	0.2 (0.20–0.27)	**313(0.5)**	**0.2 (0.2–0.3)**
45–54	635 (1.6)	1.08 (1.0–1.2)	589 (2.1)	1.0 (0.9–1.1)	**1,224 (1.8)**	**1.0 (1.0–1.1)**
55–64	3,236 (8.1)	5.3 (5.2–5.5)	2,034 (7.2)	3.20 (3.1–3.3)	**5,270 (7.8)**	**4.2 (4.1–4.4)**
65–74	9,639 (24.3)	21.1 (20.6–21.5)	5,610 (19.9)	10.9 (10.6–11.1)	**15,249 (22.5)**	**15.6 (15.4–15.9)**
≥75	25,963 (65.4)	91.9 (90.8–93.0)	19,683 (70.0)	50.1 (49.4–50.8)	**45,646 (67.3)**	**67.6 (67.0–68.2)**
**Race^††^**						
American Indian or Alaska Native	352 (0.9)	4.2 (3.2–5.1)	265 (0.9)	3.6 (3.3–4.0)	**617 (0.9)**	**3.8 (3.1–4.5)**
Asian or other Pacific Islander	1,201 (3.0)	2.8 (2.5–3.0)	928 (3.3)	2.0 (1.8–2.2)	**2,129 (3.1)**	**2.4 (2.2–2.6)**
Black or African American	1,748 (4.4)	2.6 (2.0–3.1)	1,707 (6.1)	1.4 (1.2–3.0)	**3,455 (5.1)**	**2.5 (2.3–2.7)**
White	36,253 (91.3)	8.9 (8.8–9.1)	25,103 (89.2)	7.2 (7.0–7.3)	**61,356 (90.4)**	**8.2 (8.1–8.4)**
Multiple	158 (0.4)	—	128 (0.5)	—	**286 (0.4)**	**—**
**Ethnicity**						
Hispanic or Latino	3,873 (9.8)	3.4 (3.1–3.6	3,177 (11.3)	3.2 (3.0–3.4)	**7,050 (10.4)**	**3.4 (3.2–3.6)**
Non-Hispanic	35,839 (90.2)	8.3 (8.1–8.4)	24,954 (88.7)	7.0 (6.9–7.2)	**60,793 (89.6)**	**7.7 (7.6–7.8)**

**Source:** National Vital Statistics System public use multiple cause files 2020–2022. https://www.cdc.gov/nchs/data_access/vitalstatsonline.htm#Mortality_Multiple

**Abbreviations**: ICD-10 = *International Classification of Diseases, Tenth Revision*; IPF = idiopathic pulmonary fibrosis.

* Death records with ICD-10 code J84.1 (other interstitial pulmonary diseases with fibrosis) listed as the underlying or contributing causes of death and no ICD-10 codes for any condition that might be associated with pulmonary fibrosis including underlying connective tissue diseases (M05–M08.9, M32–M35.0, M35.1, M35.5, M35.8–M36), radiation fibrosis (J70.1), sarcoidosis (D86–D86.9), pneumoconiosis (J60–J65), and hypersensitivity pneumonitis (J67–J67.9).

^†^ Age-adjusted death rates (deaths per 100,000 persons) were calculated by applying age-specific death rates to the 2000 U.S. Census Bureau standard population age distribution. https://wonder.cdc.gov/wonder/help/mcd.html#Age-Adjusted%20Rates

^§^ Decedents with information on their usual industry and occupation.

^¶^ Starting in 2020, CDC’s National Center for Health Statistics and the National Institute for Occupational Safety and Health began a collaboration to translate industry and occupation information, which was submitted by jurisdictions to National Center for Health Statistics as part of their death certificate data, to U.S. Census Bureau industry and occupation codes. Forty-seven jurisdictions participated (Arizona, North Carolina, Rhode Island, and the District of Columbia did not participate) in this program in 2020. Iowa participated in the program in 2020, but because of differences in the method of data collection, the data were inconsistent with those from other jurisdictions and were excluded. In 2021, a total of 49 jurisdictions participated (Rhode Island and the District of Columbia did not participate). All jurisdictions participated in the program in 2022. https://www.cdc.gov/nchs/data/dvs/Industry-and-Occupation-data-mortality-2020.pdf

** Age-specific IPF death rates (deaths per 100,000 persons).

^††^ Race and Hispanic origin are reported separately on the death certificate. The American Indian or Alaska Native race category includes North, Central, and South American Indians, Eskimos, and Aleuts. The Asian or other Pacific Islander race category includes Chinese, Filipino, Hawaiian, Japanese, and other Asian or other Pacific Islanders (https://wonder.cdc.gov/wonder/help/mcd.html). Race and ethnicity on death certificates might be misclassified. Persons of Hispanic or Latino (Hispanic) origin might be of any race but are categorized as Hispanic. Classification is highly accurate for both White and Black or African American populations and accurate for the Asian or other Pacific Islander and Hispanic populations. The quality of reporting for the American Indian or Alaska Native population might be poor. https://www.cdc.gov/nchs/data/series/sr_02/sr02_172.pdf

### Industry

By industry, the highest number of IPF deaths occurred among males in the manufacturing industry (7,525; 18.9% of IPF deaths in males) and among females in the health care and social assistance industry (4,277; 15.2% of IPF death in females) (Table 2). The highest significantly elevated PMRs were among males working in utilities^¶¶¶^ (1.15; 95% CI = 1.08–1.24) and public administration**** (1.15; 95% CI = 1.11–1.19) and among females working in public administration industries (1.12; 95% CI = 1.06–1.18). In addition, among both male and female workers, the elevated PMRs were found in health care and social assistance (1.11; 95% CI = 1.05–1.17 and 1.10; 95% CI = 1.07–1.13, respectively), and educational services industries (1.07; 95% CI = 1.02–1.12 and 1.09; 95% CI = 1.05–1.13).

**TABLE 2:** Industries and occupations with idiopathic pulmonary fibrosis* deaths and proportionate mortality ratio^†^ among ever-employed^§^ persons aged ≥15 years, by sex (N = 67,843) — selected U.S. jurisdictions,^¶^ 2020–2022

Characteristic	Males			Females		
	No. of deaths from all causes	IPF		No. of deaths from all causes	IPF	
		No. of deaths	PMR (95% CI)		No. of deaths	PMR (95% CI)
**Total**	**5,117,769**	**39,712**	**—**	**4,620,502**	**28,131**	**—**
**Industry**						
Accommodation and food services	163,661	773	0.84 (0.78–0.90)	193,325	885	0.85 (0.79–0.90)
Administrative and support and waste management and remediation services	160,749	864	0.87 (0.81–0.93)	69,993	419	1.05 (0.96–1.16)
Agriculture	174,627	1,386	0.87 (0.82–0.92)	34,335	248	1.08 (0.95–1.23)
Arts, entertainment, and recreation	81,121	499	0.87 (0.79–0.95)	49,635	292	0.98 (0.87–1.10)
Construction	688,686	4,553	0.94 (0.91–0.96)	32,698	205	1.06 (0.92–1.22)
Education services	200,752	1,952	1.07 (1.02–1.12)**	439,689	3,013	1.09 (1.05–1.13)**
Finance and insurance	118,280	1,181	1.10 (1.04–1.16)**	162,592	1,065	1.04 (0.98–1.11)
Health care and social assistance	166,684	1,393	1.11 (1.05–1.17)**	677,526	4,277	1.10 (1.07–1.13)**
Information	92,531	867	1.06 (0.99–1.14)	81,266	469	0.92 (0.84–1.01)
Management of companies and enterprises	6,347	62	1.03 (0.80–1.34)	7,648	46	0.93 (0.70–1.27)
Manufacturing	829,308	7,525	1.05 (1.03–1.08)**	374,764	2,331	0.98 (0.94–1.02)
Mining	66,531	600	1.04 (0.96–1.13)	3,806	26	1.08 (0.74–1.66)
Other services (except public administration)	272,497	2,060	1.00 (0.95–1.04)	186,852	1,101	1.02 (0.96–1.08)
Professional, scientific, and technical services	221,537	2,147	1.12 (1.07–1.17)**	141,938	927	1.06 (0.99–1.13)
Public administration	275,268	2,800	1.15 (1.11–1.19)**	179,848	1,220	1.12 (1.06–1.18)**
Real estate and rental and leasing	60,854	558	1.08 (1.00–1.18)**	59,421	412	1.08 (0.98–1.19)
Retail trade	352,893	2,853	1.00 (0.97–1.04)	329,300	2,009	1.01 (0.97–1.05)
Transportation and warehousing	430,807	3,154	0.95 (0.92–0.99)	81,278	455	0.98 (0.90–1.08)
Utilities	79,485	817	1.15 (1.08–1.24)**	17,266	111	1.02 (0.84–1.23)
Wholesale trade	71,483	594	0.99 (0.91–1.07)	18,889	137	1.17 (0.99–1.39)
All other industries^††^	603,668	3,074	0.85 (0.82–0.89)	1,478,433	8,483	0.92 (0.90–0.94)
**Occupation**						
Architecture and engineering	207,191	2,193	1.11 (1.06–1.16)**	15,170	91	0.97 (0.79–1.20)
Arts, design, entertainment, sports, and media	86,384	604	0.91 (0.84–0.98)	64,529	398	0.99 (0.90–1.09)
Building and grounds cleaning and maintenance	178,878	1,048	0.88 (0.83–0.94)	116,842	618	0.98 (0.90–1.06)
Business and financial operations	127,425	1,283	1.12 (1.06–1.18)**	118,245	795	1.10 (1.03–1.18)**
Community and social services	67,772	711	1.23 (1.14–1.32)**	63,789	407	1.09 (0.99–1.20)
Computer and mathematical	64,785	570	1.17 (1.08–1.27)**	21,043	106	0.88 (0.72–1.07)
Construction and extraction	643,120	4,271	0.93 (0.91–0.96)	11,205	60	1.02 (0.79–1.34)
Education, training, and library	121,243	1,243	1.08 (1.02–1.14)**	298,528	2,014	1.07 (1.02–1.12)**
Farming, fishing, and forestry	56,836	363	0.79 (0.71–0.87)	11,011	97	1.24 (1.01–1.53)**
Food preparation and serving related	108,956	399	0.78 (0.70–0.86)	176,195	819	0.84 (0.79–0.90)
Health care practitioners and technical	88,465	864	1.13 (1.06–1.21)**	277,318	2,014	1.21 (1.16–1.27)**
Health care support	13,810	58	0.82 (0.64–1.08)	152,238	770	0.97 (0.91–1.05)
Installation, maintenance, and repair	332,080	2,578	0.98 (0.95–1.02)	12,376	77	1.06 (0.85–1.34)
Legal	36,873	356	1.02 (0.91–1.13)	21,895	142	1.09 (0.92–1.29)
Life, physical, and social science	45,297	484	1.16 (1.06–1.26)**	18,111	109	0.97 (0.80–1.18)
Management	562,616	5,715	1.12 (1.09–1.15)**	250,981	1,701	1.10 (1.05–1.15)**
Office and administrative support	168,359	1,248	0.97 (0.92–1.03)	682,562	4,521	1.05 (1.02–1.08)**
Personal care and service	50,357	313	0.94 (0.84–1.05)	167,819	951	1.00 (0.93–1.06)
Production	483,066	3,994	0.99 (0.96–1.02)	257,524	1,585	0.96 (0.92–1.01)
Protective service	145,912	1,296	1.11 (1.05–1.17)**	26,955	151	1.07 (0.91–1.26)
Sales and related	374,101	3,393	1.04 (1.01–1.08)**	299,727	1,866	1.02 (0.98–1.07)
Transportation and material moving	572,375	3,577	0.89 (0.86–0.92)	106,746	556	0.93 (0.85–1.01)
All other occupations^§§^	581,868	3,151	0.88 (0.85–0.91)	1,449,693	8,283	0.92 (0.90–0.94)

**Source**: National Vital Statistics System public use multiple cause files 2020–2022. https://www.cdc.gov/nchs/data_access/vitalstatsonline.htm#Mortality_Multiple

**Abbreviations**: ICD-10 = *International Classification of Diseases, Tenth Revision*; IPF = idiopathic pulmonary fibrosis; NCHS = National Center for Health Statistics; NIOSH = National Institute for Occupational Safety and Health; PMR = proportionate mortality ratio.

* Death records with ICD-10 code J84.1 (other interstitial pulmonary diseases with fibrosis) listed as the underlying or contributing causes of death and no ICD-10 codes for any condition that might be associated with pulmonary fibrosis including underlying connective tissue diseases (M05–M08.9, M32–M35.0, M35.1, M35.5, M35.8–M36), radiation fibrosis (J70.1), sarcoidosis (D86–D86.9), pneumoconiosis (J60–J65), and hypersensitivity pneumonitis (J67–J67.9).

^†^ PMR was defined as the observed number of deaths from IPF in a specified industry, divided by the expected number of deaths from IPF. The expected number of deaths was the total number of deaths in industry of interest multiplied by a proportion defined as the number of IPF deaths in all industries, divided by the total number of deaths in all industries. The IPF PMRs were adjusted by 10-year age groups and race and ethnicity. A PMR >1.0 indicates that there were more deaths associated with IPF in a specified industry than expected.

^§^ Decedents with information on their usual industry and occupation.

^¶^ Starting in 2020, CDC’s NCHS and NIOSH began a collaboration to translate industry and occupation information which was submitted by jurisdictions to NCHS as part of their death certificate data, to U.S. Census Bureau industry and occupation codes. Forty-seven jurisdictions participated (Arizona, North Carolina, Rhode Island, and the District of Columbia did not participate) in this program in 2020. Iowa participated in the program in 2020, but because of differences in the method of data collection, the data were inconsistent with those from other jurisdictions and were excluded. In 2021, a total of 49 jurisdictions participated (Rhode Island and the District of Columbia did not participate). All jurisdictions participated in the program in 2022. https://www.cdc.gov/nchs/data/dvs/Industry-and-Occupation-data-mortality-2020.pdf

** A 95% CI lower level >1.0 indicates substantially elevated PMR.

^††^ Military, miscellaneous, or unclassifiable.

^§§^ Military, miscellaneous, unclassifiable, and homemakers.

### Occupation

By occupation, the highest number of IPF deaths occurred among males in management (5,715; 14.4% of IPF deaths in males) and among female office and administrative support workers (4,521; 16.1% of IPF deaths in females). The highest significantly elevated PMRs were among male community and social services workers (1.23; 95% CI = 1.14–1.32) and among female farming, fishing, and forestry workers (1.24; 95% CI = 1.01–1.53). Among both male and female workers, the elevated PMRs were found in the health care practitioners and technical occupations (1.13; 95% CI = 1.06–1.21 and 1.21; 95% CI = 1.16–1.27, respectively).

## Discussion

During 2020–2022, 67,843 deaths (39,712 in males and 28,131 in females) among ever-employed persons aged ≥15 years were associated with IPF. Based on an estimate that 21% of IPF deaths might be related to occupational exposures (*6*), approximately 14,248 deaths (8,340 in males and 5,908 deaths in females) might have been job-related. However, the estimate was based on non-U.S. reports and might not be directly applicable to the U.S. workforce. 

Elevated IPF death rates among persons aged ≥75 years and males are consistent with previous reports of increased mortality in these groups (*3*). In contrast to findings from a previous study, the overall age-adjusted IPF death rate of 7.1 per 100,000 persons over the 3 years of this study was higher than that reported for 2017 (5.3 per 100,000) and the rates in White and non-Hispanic persons (8.2 and 7.7 per 100,00, respectively) were higher than average annual rates reported for 2004–2017 (6.1 and 6.2 per 100,000, respectively) (*3*). These differences could be partially explained by differences in research methodologies, improved precision in diagnostic criteria, and increasing implementation of recommendations for diagnosing IPF (*1*), and differences over time in the prevalences of known IPF-associated risk factors (*6**,**7*). The decrease in cigarette smoking (*7*) (known to be associated with IPF) among adults highlights the potentially increasing proportionate role of environmental and occupational exposures in the development of IPF (*4**–**6*).

Higher proportions of IPF deaths were observed among ever-employed persons in several industries and occupations. Among both male and female workers, the highest significantly elevated PMRs were found in the public administration, health care and social assistance, and educational services industries as well as in the health care practitioners and technical occupations. Workers in some of these industries and occupations would be anticipated to have frequent exposure to secondhand smoke^††††^ (*8*); vapors, gas, dust, and fumes^§§§§^ (*8*); biologic (e.g., bioaerosols in indoor environments) (*2**,**9**,**10*); chemical (e.g., pesticides) (*5*); and other hazards in the workplace (*5**–**10*). However, for some industries and occupations at increased risk, potential sources of increased risk are unclear and might be related to either work exposures or factors not directly related to work that were not fully addressed by the study design.

### Limitations

The findings in this report are subject to at least eight limitations. First, no ICD-10 code is specific to IPF, and IPF might be underreported on death certificates (*3*). Second, the IPF diagnosis might be affected by access to specialty care and chest computed tomography, both to identify interstitial lung disease and exclude other known causes of interstitial lung disease. However, access to care information was not available, and IPF deaths were not validated using medical records. Third, because of the cross-sectional study design, no temporal relationship between IPF death and work could be measured. Fourth, information on smoking status and workplace exposures are not recorded on death certificates. Therefore, these exposures could not be evaluated for their association with IPF deaths. Fifth, the decedent’s usual industry and occupation information reported on the death certificate might not be the industry and occupation associated with IPF deaths. No work histories are recorded on death certificates to evaluate changes in employment. Sixth, multiple comparisons might identify industries and occupations with elevated PMR by chance and thus these might not actually represent occupational risk. Sixth, small sample sizes for some groups resulted in wide PMR CIs. Seventh, application of a population attributable fraction estimate from a different study population to these data is speculative and should be interpreted with caution. Finally, this was an exploratory analysis with no guiding hypotheses; therefore, these findings should be considered hypothesis-generating.

### Implications for Public Health Practice

Estimates of elevated IPF mortality among ever-employed persons in certain industries and occupations suggest areas where targeted studies, and processes to identify and control causative workplace exposures according to the applicable standard might be considered.^¶¶¶¶^ Primary prevention would involve using the hierarchy of controls (elimination, substitution, engineering controls, administrative controls, and personal protective equipment) to reduce or eliminate exposures to potentially causative work hazards.***** In addition, smoke-free workplace policies and tobacco cessation programs can help reduce or eliminate exposure to tobacco smoke. Continued research to confirm these findings, and surveillance including collection of detailed industry and occupational history and etiologic research to further characterize occupational risk factors for IPF, are essential to guide development and implementation of evidence-based interventions and policies to improve workers’ health.

## Footnotes

* The estimate was made in a meta-analysis using data from 11 studies from the following countries: United Kingdom (three), South Korea (two), Japan (one), Sweden (one), Mexico (one), Italy (one), Australia (one), and Pakistan (one). Applicability of the estimate to U.S. workers should be interpreted with caution.

†
https://www.cdc.gov/nchs/data_access/vitalstatsonline.htm#Mortality_Multiple

§ Forty-seven jurisdictions submitted industry and occupation information (Arizona, North Carolina, Rhode Island, and the District of Columbia did not) in 2020, a total of 49 jurisdictions (Rhode Island and the District of Columbia did not) in 2021. Iowa participated in the program in 2020, but because of differences in the method of data collection, the data were inconsistent with those from other jurisdictions and were excluded. All jurisdictions participated in 2022. Foreign residents, decedents aged <15 years, persons with missing age, and decedents from the nonparticipating or excluded jurisdictions were not included in industry and occupation coding, resulting in 9,738,271 records. https://ftp.cdc.gov/pub/Health_Statistics/NCHS/Dataset_Documentation/DVS/mortality/Industry-and-Occupation-data-mortality-2020.pdf

¶ Industry (the business activity of a person’s employer or, if self-employed, their own business) and occupation (a person’s job or the type of work they do) are used to categorize employment. Usual occupation and industry recorded on the death certificate is the occupation and industry in which the decedent spent a majority of their working life.

**
https://www.bls.gov/cps/definitions.htm#occupation

†† The underlying cause of death is the disease or injury that initiated the chain of events that led directly and inevitably to death. https://www.cdc.gov/nchs/icd/icd-10-cm/

§§ Underlying connective tissue diseases: M05–M08.9, M32–M35.0, M35.1, M35.5, M35.8–M36; radiation fibrosis: J70.1; sarcoidosis: D86–D86.9; pneumoconiosis: J60–J65; and hypersensitivity pneumonitis: J67–J67.9.

¶¶ For calculation of death rates, numerators were the sum of IPF deaths that occurred during 2020–2022, and denominators were the sum of 2020, 2021, and 2022 midpoint populations (for 2020 and 2021 population data for jurisdictions that did not provide industry and occupation information were removed from denominators). Age-adjusted death rates were calculated by applying age-specific death rates to the 2000 U.S. Census Bureau standard population age distribution. https://wonder.cdc.gov/wonder/help/mcd-expanded.html#Age-Adjusted%20Rates*** https://www.dol.gov/agencies/wb/data/Employment-and-Earnings-by-Occupation

††† PMR was defined as the observed number of deaths from IPF in a specified industry or occupation, divided by the expected number of deaths from IPF. The expected number of deaths was the total number of deaths in an industry or occupation of interest multiplied by a proportion defined as the number of IPF deaths in all industries or occupations, divided by the total number of deaths in all industries or occupations. The IPF PMRs were adjusted by 10-year age groups, sex, race, and ethnicity.

§§§ 45 C.F.R. part 46; 21 C.F.R. part 56; 42 U.S.C. Sect. 241(d), 5 U.S.C. Sect. 552a, 44 U.S.C. Sect. 3501 et seq.

¶¶¶ Of all 364 occupations in the utilities industry, 119 were associated with IPF deaths; four occupations had 50 or more IPF deaths and include electrical power-line installers and repairers; managers, all other; first-line supervisors of production and operating workers; and electricians.

**** Of all 497 occupations in the public administration industry, 263 were associated with IPF deaths; 16 occupations had 50 or more IPF deaths and include police and sheriff’s patrol officers; managers, all other; secretaries and administrative assistants; office clerks, general; firefighters; supervisors of protective service workers, all other; accountants and auditors; first-line supervisors of police and detectives; detectives and criminal investigators; social workers; compliance officers; engineers, all other; janitors and building cleaners; general and operations managers; and maintenance and repair workers, general.

†††† For example, the proportions of nonsmoking workers exposed to secondhand smoke in the utilities industry, public administration industry and health care and social assistance industry have been estimated at 15.2%, 10.5%, and 8.0%, respectively; these proportions among health care practitioners and technical workers, and health care support workers have been estimated at 6.3% and 11%, respectively.

§§§§ For example, the proportion of workers reporting frequent exposure to vapors, gas, dust, or fumes at work in the public administration industry and health care and social assistance industry has been estimated at 21.6% and 13.4%, respectively; this proportion among health care practitioners and technical workers, and health care support workers has been estimated at 14.2% and 15.0%, respectively.

¶¶¶¶
https://www.osha.gov/laws-regs

*****
https://www.cdc.gov/niosh/hierarchy-of-controls/about/index.html
